# P-1081. Activity of Aztreonam-Avibactam against Enterobacterales Isolated from Patients with Intra-Abbdominal Infection from United States Medical Centers (2019-2023)

**DOI:** 10.1093/ofid/ofae631.1269

**Published:** 2025-01-29

**Authors:** Helio S Sader, Timothy Doyle, Rodrigo E Mendes, Marisa Winkler, Mariana Castanheira

**Affiliations:** JMI Laboratories, North Liberty, Iowa; Element Materials Technology/Jones Microbiology Institute, North Liberty, Iowa; JMI Laboratories, North Liberty, Iowa; Element Materials Technology/Jones Microbiology Institute, North Liberty, Iowa; JMI Laboratories, North Liberty, Iowa

## Abstract

**Background:**

Aztreonam-avibactam (ATM-AVI) is being developed for the treatment of Gram-negative infections, including those caused by metallo-β-lactamase (MBL) producers. We evaluated the activity of ATM-AVI against Enterobacterales (ENT) isolated from patients with intraabdominal infections (IAI) in United States (US) medical centers.

Activity of β-lactamase inhibitor combinations against Enterobacterales from intra-abdominal infections

**Figure:**
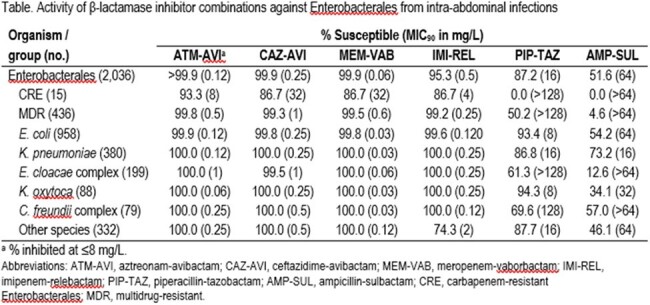

**Methods:**

2,036 isolates (1/patient) were consecutively collected from patients with IAI in 63 US hospitals in 2019-2023. Susceptibility testing was performed by broth microdilution method. ATM-AVI was tested with AVI at fixed 4 mg/L and a pharmacokinetic/pharmacodynamic susceptible (S) breakpoint of ≤8 mg/L was applied for comparison. Carbapenem-resistant ENT (CRE) were screened for carbapenemases (CBase) by whole genome sequencing. Comparator agents included ceftazidime-avibactam (CAZ-AVI), meropenem-vaborbactam (MEM-VAB), imipenem-relebactam (IMI-REL), and cefiderocol (CRE only), among others.

**Results:**

The most common ENT species were *E. coli* (47.1%), *K. pneumoniae* (18.7%), and *E. cloacae* species complex (9.8%). Only 1 ENT exhibited ATM-AVI MIC >8 mg/L (MIC_50/90_, ≤0.03/0.12 mg/L), an *E. coli* with ATM-AVI MIC of 16 mg/L. The most active agents against CRE were ATM-AVI (93.3% inhibited at ≤8 mg/L) and cefiderocol (93.3% S); CAZ-AVI, MEM-VAB, and IMI-REL were active against 86.7% of CRE (Table). A multidrug-resistant (MDR) phenotype (non-S to ≥3 classes) was observed in 21.4% of ENT (*n*=436) and the most active β-lactamase inhibitor combination (BLIC) against MDR isolates were ATM-AVI (99.8% inhibited at ≤8 mg/L), MEM-VAB (99.5% S), and CAZ-AVI (99.3% S). Piperacillin-tazobactam (PIP-TAZ) was active against 87.2% of ENT and 50.2% of MDR, and meropenem was active against 92.2% of ENT and 96.1% of MDR. A CBase was identified in 9 isolates (60.0% of CRE), and included KPC-2 (3 isolates), KPC-3 (4), and NDM-5 (2). All CBase producers were inhibited at ≤8 mg/L of ATM-AVI and were cefiderocol-S; whereas susceptibility to CAZ-AVI, MEM-VAB, and IMI-REL were 77.8%.

**Conclusion:**

ATM-AVI and the newer BLICs exhibited almost complete activity against ENT causing IAI in US hospitals. ATM-AVI may represent a valuable treatment option for IAI, including those caused by CRE and MDR ENT.

**Disclosures:**

**Rodrigo E. Mendes, PhD**, GSK: Grant/Research Support **Marisa Winkler, MD, PhD**, Element Iowa City (JMI Laboratories) was contracted to perform services in 2023 for > 30 biotech and pharmaceutical companies: Grant/Research Support

